# RACIAL DISPARITIES IN PSYCHOLOGICAL WELL-BEING ACROSS AGE GROUPS DURING COVID-19: A LONGITUDINAL STUDY

**DOI:** 10.1093/geroni/igae098.2338

**Published:** 2024-12-31

**Authors:** Meghan Elliott, Susan Charles, Roxane Cohen Silver, E Alison Holman

**Affiliations:** University of California Irvine, Irvine, California, United States; University of California Irvine, Irvine, California, United States; University of California Irvine, Irvine, California, United States; University of California Irvine, Irvine, California, United States

## Abstract

COVID-19 created a health threat that discriminated by age: older adults were at greatest risk for health complications and mortality, but younger adults reported the highest rates of emotional distress. Cross-sectional studies consistently documented age differences in distress, but few studies have examined age-related differences over time. We examined how self-reported anger and both depressive and anxiety symptoms changed throughout the first two years of the COVID-19 pandemic among a national representative sample of adults (N = 6514) aged 18 to 97 years who completed four surveys between March 2020 and June 2022. Multi-level models revealed age differences that varied by race. Overall, younger age and non-white status were related to higher levels of every affective distress measure. For depressive symptoms, most age groups peaked at wave 2 and declined thereafter. The exception was the young non-white adults, where levels peaked at wave 2 and remained stable thereafter. For most participants, anxiety symptoms were highest at wave 1 and declined thereafter, although young non-white participants had minimal decreases and levels increased among middle-aged and older adults across race. Anger symptoms peaked for all age groups at wave 2, yet older white adults were the only group whose anger levels did not decrease from wave 2 to wave 4. Results suggest that non-white status and young age may be vulnerabilities to symptoms of depression and anxiety, and being old and white is related to stable levels of anger across time, highlighting the need for targeted support for these groups.

